# Vitamin D Deficiency among Newborns in Amman, Jordan

**DOI:** 10.5539/gjhs.v6n1p162

**Published:** 2013-11-06

**Authors:** Najwa Khuri-Bulos, Ryan D. Lang, Meridith Blevins, Katherine Kudyba, Lindsey Lawrence, Mario Davidson, Samir Faouri, Natasha B. Halasa

**Affiliations:** 1 Division of Pediatric Infectious Disease, Department of Pediatrics, Vanderbilt University School of Medicine; Nashville, TN, USA; 2 Department of Medicine, Johns Hopkins University School of Medicine; Baltimore, MD, USA; 3 Department of Biostatistics, Vanderbilt University School of Medicine; Nashville, TN, USA; 4 Division of Infectious Diseases, Department of Pediatrics, University of Jordan School of Medicine, Amman, Jordan; 5 Department of Emergency Medicine, Mount Sinai, New York, NY, USA; 6 Department of Pediatrics, Al Bashir Government Hospital, The Jordan Ministry of Health, Amman, Jordan

**Keywords:** Jordan, vitamin D deficiency, newborn, infant, Middle East

## Abstract

**Objective:**

Vitamin D deficiency is well recognized in selected Middle Eastern countries, but neonatal vitamin D status is not well studied in Jordan and other nearby countries. The aim of this study is to determine the prevalence of vitamin D deficiency in Jordanian newborns and risk factors associated with low levels.

**Methods:**

This is a prospective cohort study of newborn infants who were delivered at the Al Bashir Government Hospital in Amman, Jordan, from January 31, 2010, to January 27, 2011. Heel stick blood samples for 25-hydroxyvitamin D [25(OH)D] levels were obtained within 96 hours of birth. Maternal dress pattern, vitamin supplementation, smoke exposure during pregnancy, mode of delivery, gestational age, and birth weight were documented.

**Results:**

Samples were obtained from 3,731 newborns. Median gestational age was 39 weeks, median birth weight was 3.1 kilograms, median maternal age was 27 years, and median newborn 25(OH)D level was 8.6nmol/L. A total of 3,512 newborns (94.1%) in this study were vitamin D deficient (< 50 nmol/L). Lower gestational age, maternal smoke exposure, and birth during winter months were associated with lower infant vitamin D levels, while vitamin D supplementation and time spent outside during pregnancy were associated with higher vitamin D levels.

**Conclusions:**

The prevalence of severely low vitamin D levels in newborn infants in Amman, Jordan, is substantial, even in newborns born during the spring and summer months. Vitamin D supplementation is needed in this population.

## 1. Introduction

Vitamin D is essential for the maintenance of bone structure and support. In its active form (1,25-dihydroxyvitamin D), vitamin D potentiates the absorption of calcium and phosphate from the small intestine into the circulatory system (DeLuca, 2004; Demay et al., 2007; Holick, 2008b). The primary source of vitamin D for humans is sunlight; nutritional sources of vitamin D are few, and they include vitamin D-fortified milk, eggs, liver, cod oil, fatty fish (e.g. salmon, tuna), and other fish oils (Holick, 2008b). Vitamin D deficiency, defined in this study as 25-hydroxyvitamin D [25(OH)D] concentration less than 50 nmol/L (<20 ng/mL), has been associated with interrupted bone growth and/or ossification, growth retardation, dysfunctional calcium metabolism, and other adverse health effects (Hollis & Wagner, 2005; Holick, 2008b; a). Other studies have described the association of vitamin D deficiency and increased risk for infants to develop type 1 diabetes mellitus, cancers, and other endocrine disorders (Hypponen et al., 2001; Hollis & Wagner, 2005; Holick, 2008a). The association of vitamin D deficiency with increased severity of respiratory infections has also been reported, and its possible association with asthma is an evolving issue (Roth et al., 2008; Ginde et al., 2009).

The major consequence of vitamin D deficiency in children is rickets, which manifests as skeletal malformation and growth deprivation. Common risk factors for developing rickets include dark skin pigmentation, inadequate sunlight, and insufficient vitamin D supplementation with breast milk (Mughal, 2011). Neonatal vitamin D levels are dependent on the maternal vitamin D status at delivery, and maternal vitamin D levels and newborn vitamin D levels are directly correlated or even lower concentrations in newborns compared to mothers (Bouillon et al., 1977; Bouillon et al., 1981; Hollis & Wagner, 2004). It is hypothesized that 25(OH)D and 24,25(OH)D freely diffuse across the placenta during pregnancy due to the similar concentrations of these substances in the fetus and the mother (Salle et al., 2001; 2005). Low maternal vitamin D levels are also associated with a higher prevalence of respiratory infections in newborns and wheezing episodes later in life (Gale et al., 2008). Still, the long-term consequences of vitamin D deficiency for newborns are not fully understood.

Vitamin D deficiency is a prominent health issue worldwide and the severity of vitamin D deficiency is significant in developing countries, in particular in Middle Eastern countries. An increased prevalence of vitamin D deficiency has been documented in Saudi Arabia, Lebanon, Jordan, and Iran; however, studies from Jordan and other countries in the region did not include a large group of newborns and/or pregnant women (Salimpour, 1975; Fuleihan & Deeb, 1999; Gannage-Yared et al., 2000; Dawodu et al., 2001; Dawodu et al., 2005). Therefore, the current study was conducted in order to determine the status of vitamin D levels in a large Jordanian newborn cohort born over the different seasons at a government hospital and to determine associations with low vitamin D levels over a 12-month period. In addition, other risk factors for caesarean section, NICU admission, and low birth weight were also explored in this cohort.

## 2. Methods

### 2.1 Study Design

Infants born at Al Bashir Government Hospital were prospectively enrolled at birth for a one-time assessment of their vitamin D levels. Verbal consent was obtained from mothers to obtain heel sticks for blood from their infants. Mothers were informed of the risks and benefits of the study and explained how data from blood samples would be used. A brief questionnaire assessing demographic and social behaviors of the mothers was used. All infants enrolled within 96 hours of birth were eligible for enrollment. Al Bashir Government Hospital is one of three major hospitals serving Amman, Jordan. This study was approved by the University of Jordan and the institutional review boards of Vanderbilt University and the Jordanian Ministry of Health at Al Bashir Hospital.

### 2.2 Vitamin D Samples

After a heel stick was performed, blood was directly placed on filter paper and air dried for at least 30 minutes before storage at room temperature and kept in a dry state until shipment to ZRT Laboratory (Beaverton, OR) for vitamin D assay per protocol (Newman et al., 2009; Larkin et al., 2011). One 6 mm blood spot is punched for each sample and control. Samples are extracted in deionized water with sonication. Proteins are precipitated in methanol, at which time internal standard (D3-25-hydroxyvitamin D3) is added, and centrifuged. Supernatant is applied to solid phase extraction (C18) columns and eluted with ethyl acetate. Extracts are derivatized with PTAD (4-Phenyl-1,2,4-triazoline-3,5-dione, Sigma), dried, and reconstituted with 50:50 methanol:deionized water. Twenty microliters (20 μL) of the final solution are injected and analyzed by liquid chromatography tandem mass spectrometry (Varian). The 25(OH)D (vitamin D) levels were reported as nanomols per liter (nmol/L).

### 2.3 Questionnaires

Using a standardized case report questionnaire, mothers were queried in Arabic by a member of the research team, who subsequently recorded the answers in English. Parents were asked to provide nationality of mother and father, child’s date of birth, route of delivery, child’s birth weight, mother’s vitamin D supplementation history, daily number of hours that mother spends outdoors, mother’s clothing practice, whether or not the mother smoked during pregnancy (and if so, which of the trimesters), and if the mother was exposed to smoke in her household during pregnancy. Mothers were screened for medical conditions that may be associated with decreased bone health including hyperparathyroidism, gestational diabetes, rheumatoid arthritis, and diseases requiring corticosteroid treatment. Data were entered into a secured electronic database (Vanderbilt RedCAP) (Harris et al., 2009).

### 2.4 Statistical Analysis

Descriptive statistics summarized patient characteristics by median vitamin D level. A multivariable linear regression model was used to assess the relationship between hypothesized predictors and vitamin D level. Covariates of interest included: sex, gestational age, maternal age, smoking exposure, supplementation, sun exposure, mother's dress, and birth date. A marginal effects plot demonstrated the covariate-adjusted relationship between date of birth and vitamin D in order to investigate seasonality. We employed multivariable logistic and linear regression models to assess the independent association between vitamin D and 3 birth outcomes: Caesarean section, neonatal intensive care unit (NICU) admission, and birth weight. We adjusted for sex, gestational age, maternal age, and birth date. Birth weight was log-transformed to satisfy model assumptions. In all models, missing values of predictors were accounted for using multiple imputation techniques. We used the functions ‘aregImpute’ and ‘fit.mult.impute’ from the Hmisc package in R which used predictive mean matching to take random draws from imputation models; 25 imputation data sets were used in the analysis. When linearity assumptions were violated, we modeled continuous variables using a restricted cubic spline with 4 or more knots. R software 2.13.1 (available at: http://www.r-project.org) was used for all data analyses. Analysis scripts are available at http://biostat.mc.vanderbilt.edu/ArchivedAnalyses.

## 3. Results

Of the 4,332 mothers approached, 3,889 newborns were enrolled prospectively between January 31, 2010 and January 31, 2011. Adequate samples were collected from 3,731 neonates: 1,860 females (49.9%) and 1,871 males (50.1%). The median age of mothers was 27 years, median gestational age was 39 weeks, and the median birth weight was 3.1 kg (Table 1). A total of 183 newborns (4.9%) were admitted to the NICU. Notably, 7.6% of mothers reported a history of smoking while pregnant, while 72.4% of all mothers reported secondary smoke exposure during pregnancy. A multivitamin was taken by 16.1% of mothers in the study, and 17.0% of mothers reported vitamin D supplementation during their pregnancy. Other supplements taken included: folic acid (32.2%), iron (31.9%), and calcium (20.1%). The disease most reported in mothers was rheumatoid arthritis (3.1%).

**Table 1:** Summary of newborn/mother characteristics by median infant 25(OH)D level

	25(OH)D<8.6 nmol/L (n=1865)	25(OH)D≥8.6 nmol/L (n=1866)	Combined (n=3731)
Female, n(%)	926 (49.7%)	934 (50.1%)	1860 (49.9%)
Gestational age, weeks	39 (38, 40)	39 (38, 40)	39 (38, 40)
Birth weight	3.1 (2.9, 3.5)	3.1 (2.9, 3.5)	3.1 (2.9, 3.5)
Type of birth delivery, n(%)			
Normal spontaneous vaginal delivery	1596 (85.6%)	1522 (81.6%)	3118 (83.6%)
C-Section	268 (14.4%)	344 (18.4%)	612 (16.4%)
Newborn admitted to the NICU, n(%)	88 (4.7%)	95 (5.1%)	183 (4.9%)
Age of Mother	26.7 (22.4, 32.2)	26.7 (22.5, 32.3)	26.7 (22.5, 32.2)
BMI of Mother	23.9 (21.2, 27)	23.4 (21.5, 26.7)	23.7 (21.3, 26.8)*
Mother’s highest education level, n(%)			
Primary education	208 (33.7%)	188 (32.0%)	396 (32.9%)
Preparatory school	35 (5.7%)	25 (4.3%)	60 (5.0%)
Secondary education	282 (45.7%)	271 (46.1%)	553 (45.9%)
Some college	2 (0.3%)	3 (0.5%)	5 (0.4%)
College	55 (8.9%)	63 (10.7%)	118 (9.8%)
University	15 (2.4%)	25 (4.3%)	40 (3.3%)
No education	20 (3.2%)	13 (2.2%)	33 (2.7%)
Mother’s clothing practice, n(%)			
European dress	7 (0.4%)	12 (0.6%)	19 (0.5%)
Head scarf only	1645 (88.2%)	1666 (89.3%)	3311 (88.7%)
Fully covered	213 (11.4%)	188 (10.1%)	401 (10.7%)
Mother’s Nationality, n(%)			
Jordanian	1623 (87.0%)	1596 (85.6%)	3219 (86.3%)
Egyptian	25 (1.3%)	72 (3.9%)	97 (2.6%)
Palestinian	189 (10.1%)	166 (8.9%)	355 (9.5%)
Syrian	20 (1.1%)	15 (0.8%)	35 (0.9%)
Other	8 (0.4%)	16 (0.9%)	24 (0.6%)
Father’s Nationality, n(%)			
Jordanian	1633 (87.6%)	1622 (86.9%)	3255 (87.2%)
Egyptian	32 (1.7%)	76 (4.1%)	108 (2.9%)
Palestinian	185 (9.9%)	151 (8.1%)	336 (9.0%)
Syrian	14 (0.8%)	6 (0.3%)	20 (0.5%)
Other	1 (0.1%)	11 (0.6%)	12 (0.3%)
Primary exposure to smoking, n(%)	154 (8.3%)	130 (7.0%)	284 (7.6%)
Secondary exposure to smoking, n(%)	1384 (74.2%)	1316 (70.5%)	2700 (72.4%)
Any exposure to smoking, n(%)	1406 (75.4%)	1333 (71.4%)	2739 (73.4%)
Where mother attended prenatal care^1^, n(%)			
Jordanian Ministry of Health	226 (12.1%)	239 (12.8%)	465 (12.5%)
United Nations Relief and Works Agency	267 (14.3%)	287 (15.4%)	554 (14.8%)
Private Sector	443 (23.8%)	434 (23.3%)	877 (23.5%)
University	3 (0.2%)	6 (0.3%)	9 (0.2%)
Other None specified	5 (0.3%) 1077(57.7%)	9 (0.5%) 1068(57.2%)	14 (0.4%) 2145(57.5%)

Median 25(OH)D level was 8.6 nmol/L. Percentages are computed using the number of newborns with a non-missing value. Continuous variables are reported as median with interquartile range (ie, the 25th and 75th percentiles).

1 Mothers were asked to select all that apply.

* represents only 21.8% of the mothers.

**Figure 1:**
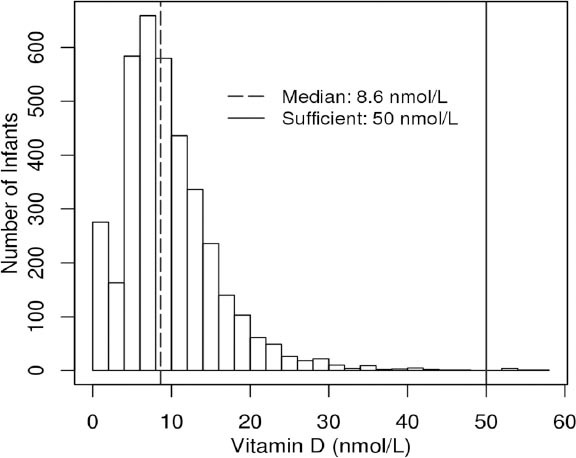
Histogram of infant 25(OH)D levels

Of the 3,731 newborns who had vitamin D levels measured, 3,512 (94.1%) had 25(OH)D levels below 50 nmol/L, with with a median of 8.6 nmol/L. Figure 1 illustrates the distribution of vitamin D levels of the study participants. The median 25(OH)D level among the infants in the study was 8.6 nmol/L. Table 1 provides a comparison of additional demographic information between those infants below and above the median 25(OH)D level. A greater frequency of caesarean sections were performed in infants whose vitamin D levels were above the median (18.0% versus 14%, p<0.001).

Graph showing distribution of newborns by measured 25(OH)D level. The hashed line represents the median vitamin D level, and the solid line represents the cut-off for 25(OH)D deficiency (<50 nmol/L). Among 3,731 newborns enrolled in the study, 3,512 (94.1%) neonates had deficient 25(OH)D levels.

**Figure 2:**
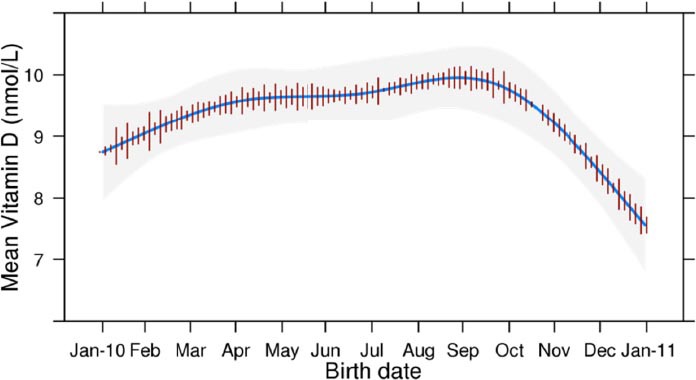
Trend of mean infant vitamin D concentration by calendar month

Estimates of independent associations between infant 25(OH)D level and pre-selected factors are shown in Table 2. Lower gestational age, maternal smoke exposure, and birth during winter months were associated with lower infant vitamin D levels, while vitamin D supplementation and time spent outside during pregnancy were associated with higher vitamin D levels. Figure 2 displays the mean infant 25(OH)D level variation over a one-year time period by date of birth.

**Table 2:** Linear regression model: infant vitamin D among newborns, Amman, Jordan, 2010-2011

	Estimate (95% CI) (nmol/L)	P-value
Female	0.23 (-0.17, 0.63)	0.255
Gestational age (per 1 month)	-0.19 (-0.30, -0.08)	<0.001*
Maternal age (per 1 year)	-0.05 (-0.21, 0.11)	0.532
Exposed to smoking	-0.72 (-1.17, -0.27)	0.002*
Any vitamin D supplementation	0.71 (0.10, 1.32)	0.022*
Time spent outside (per hour)	0.24 (0.04, 0.43)	0.019*
Mother’s clothing practice		0.187
Head scarf only (ref)	0	
European dress	2.58 (-0.23, 5.39)	
Fully covered	-0.09 (-0.74, 0.56)	
Month of birth		<0.001*
January 2010	-1.14 (-1.92, -0.35)	
February 2010	-0.83 (-1.44, -0.22)	
March 2010	-0.52 (-1.17, 0.13)	
April 2010	-0.31 (-1.01, 0.38)	
May 2010	-0.23 (-0.78, 0.31)	
June 2010	-0.22 (-0.58, 0.14)	
July 2010	-0.15 (-0.40, 0.09)	
August 2010 (ref)	0	
September 2010	0.07 (-0.24, 0.39)	
October 2010	-0.12 (-0.64, 0.40)	
November 2010	-0.66 (-1.20, -0.12)	
December 2010	-1.46 (-2.06, -0.86)	
January 2011	-2.34 (-3.19, -1.49)	

Graph shows the mean infant vitamin D level (nmol/L) by date of birth from January 2010 to January 2011. Mean vitamin D level decreases during winter months are likely due to less sunshine during these months. Estimates of mean infant vitamin D are adjusted to sex=Male, Maternal age=27 years, Smoke exposure=Yes, Vitamin D Supplementation=No, Gestational age=40 weeks, Time spent outside=1 hour, and Maternal dress=Head scarf only.

Estimates of independent associations between infant 25(OH)D level and caesarean section, NICU admission, and birth weight, while adjusting for known risk factors, are shown in Table 3. After modeling the probability of caesarean section, higher infant 25(OH)D level, gestational age (<38 weeks and ≥42 weeks), older maternal age (≥30 years), and birth month were all statistically significant predictors for caesarean section (Table 3). We did not detect an association between gender and delivery method. An independent association between infant 25(OH)D level and NICU admission or birth weight were not noted (Table 3). However, associations between gestational age (<38 weeks and ≥42 weeks) and birth months (March-May and September-November) with NICU admission were detected. In addition, male gender, older gestational age (≥42 weeks), older maternal age, and birth months (March-July, January 2011) were associated with lower infant birth weight (Table 3).

**Table 3:** Logistic regression model: caesarean section, NICU admission, and low birth weight among newborns, Amman, Jordan, 2010-2011

	Caesarean Section		NICU Admission		Log(Birth Weight)	
	Odds Ratio (95% CI)	P-value	Odds Ratio (95% CI)	P-value	Odds Ratio (95% CI)	P-value
Infant Vitamin D (nmol/L)	1.03 (1.01, 1.04)	<0.001*	0.98 (0.95, 1.01)	0.150	1.00 (1.00, 1.00)	0.088
Female	0.90 (0.74, 1.08)	0.261	0.90 (0.64, 1.26)	0.549	0.96 (0.95, 0.97)	<0.001*
Gestational age (weeks)		<0.001*		<0.001*		<0.001*
34	4.49 (3.27, 6.16)		45.21 (27.22, 75.10)		0.74 (0.72, 0.75)	
36	3.44 (2.67, 4.43)		10.14 (6.20, 16.58)		0.84 (0.83, 0.85)	
38	2.35 (1.85, 2.99)		2.44 (1.51, 3.97)		0.94 (0.93, 0.95)	
40 (ref)	1		1		1	
42	1.67 (1.06, 2.63)		1.85 (0.71, 4.79)		1.03 (1.00, 1.05)	
Maternal age (years)		<0.001*		0.331		<0.001*
20 (ref)	1		1		1	
30	2.57 (1.92, 3.45)		1.14 (0.88, 1.47)		1.02 (1.01, 1.03)	
40	4.57 (3.26, 6.40)		1.29 (0.77, 2.15)		1.04 (1.02, 1.06)	
Month of birth		<0.001*		0.022		<0.001*
Jan 2010	2.07 (1.46, 2.92)		0.69 (0.36, 1.30)		0.98 (0.96, 0.99)	
Feb 2010	0.35 (0.26, 0.47)		0.92 (0.56, 1.51)		0.99 (0.98, 1.01)	
Mar 2010	0.07 (0.04, 0.10)		1.20 (0.70, 2.06)		1.01 (0.99, 1.03)	
Apr 2010	0.03 (0.02, 0.05)		1.30 (0.72, 2.34)		1.02 (1.00, 1.04)	
May 2010	0.05 (0.04, 0.08)		1.09 (0.70, 1.71)		1.03 (1.01, 1.04)	
Jun 2010	0.20 (0.16, 0.25)		0.85 (0.65, 1.12)		1.02 (1.01, 1.03)	
Jul 2010	0.66 (0.59, 0.73)		0.79 (0.65, 0.96)		1.01 (1.01, 1.02)	
Aug 2010 (ref)	1		1		1	
Sep 2010	0.93 (0.81, 1.07)		1.34 (1.04, 1.73)		0.99 (0.98, 1.00)	
Oct 2010	0.79 (0.62, 0.99)		1.45 (0.95, 2.22)		0.99 (0.97, 1.00)	
Nov 2010	0.77 (0.61, 0.98)		1.10 (0.70, 1.73)		0.99 (0.98, 1.00)	
Dec 2010	0.85 (0.66, 1.08)		0.64 (0.36, 1.12)		1.00 (0.99, 1.02)	
Jan 2011	0.97 (0.69, 1.35)		0.34 (0.15, 0.77)		1.02 (1.00, 1.04)	

An association between infant 25(OH)D level and gestational diabetes was investigated; but the number of mothers in the study with gestational diabetes (n=11) was insufficient to complete a multivariable logistic regression. However, the test of association between infant 25(OH)D level and gestational diabetes did fall short of statistical significance (p=0.068).

## 4. Discussion

In our large, one-year prospective cross-sectional study which included 3,731 newborns, we discovered that 94% of our Jordanian newborns had vitamin D levels lower than 50 nmol/L, with a median of 8.6 nmol/L. These results correlate with other studies, which report vitamin D deficiency in Jordan and nearby countries such as Iran and Lebanon (Fuleihan & Deeb, 1999; Bassir et al., 2001). A study conducted in Jordan revealed that 37-73% of adult women and 5-22% of adult men in Jordan were vitamin D deficient compared to the National Micronutrient Survey in Jordan 2010 which revealed 60.3% of women were vitamin D deficient (Batieha et al., 2011). However, the degree of vitamin D deficiency in our population was more pronounced compared to the other studies. For instance, in a study conducted in Jordanian children aged 1-6 years, 16.5% of the children had vitamin D deficiency (serum 25 (OH) D_3_ ≤15 ng/mL), while 15.5% had vitamin D insufficiency (serum 25 (OH) D_3_ from 15 to 20 ng/mL) (Jazar, 2001). Another study of 275 Jordanian infants and toddlers aged 6-26 months (136 infants and 139 toddlers) reported that 28% of the subjects were deficient in vitamin D (16.7% for severe vitamin D deficiency and 11.3% for vitamin D deficiency) (Abdul-Razzak et al., 2011). Our study is unique, because unlike other studies, our cohort focused on newborn children.

We identified several potential risk factors for vitamin D deficiency in our population including minimal exposure to sunlight, covered maternal clothing habits, and lack of vitamin supplementation. Jordan is considered a sunny country with ample exposure to sunlight, much like nearby countries; however, our newborn cohort still had significant vitamin D deficiency, even during the summer months. The majority of our Jordanian women reported Hijab use (89%) or full covering from head to toe in a Neqab (11%), which has also been reported to be associated with lower vitamin D levels (Batieha et al., 2011).

Confounding the effects of lower vitamin D levels, many of these women do not work outside of their homes, thus making sunshine less common. Moreover, in our study population, the average reported time spent outside was only one hour. In addition, only 17% of mothers reported vitamin D supplementation during pregnancy. It is interesting to note that vitamin D supplementation during pregnancy resulted in small increases in infant vitamin D levels (0.71 nmol/L with 95% CI 0.10 to 1.32). We may speculate that the disappointingly low vitamin D levels in infants of mothers who received vitamin D supplementation may be due to irregularities in administration of vitamin D during pregnancy. Also, the self-reporting nature of the study may have affected the validity of the results as some mothers may not have received the amount of vitamin D supplementation as reported. Of note, since food in Jordan was not fortified with vitamin D during this time period, this may further contribute to low vitamin D levels. Therefore, simple interventions for pregnant women which could lead to increased vitamin D levels include: increasing their daily sun exposure throughout their pregnancy, consumption of vitamin D-fortified food, and ensuring adequate vitamin D supplementation.

We examined if caesarean delivery, NICU admission, and low birth weight were associated with low infant vitamin D levels. Our results revealed a 3% increase in the odds of Caesarean section per 1 mmol/L change in infant vitamin D level. While there is a very small effect size, this positive association is in contrast to a recent study, which reported first-trimester maternal vitamin D levels were similar between mothers who delivered vaginally and mothers who delivered by caesarean section (Savvidou et al., 2012). These conflicting correlations should be further evaluated. In contrast, we did not detect an association of vitamin D deficiency with NICU admission or low birth weight.

Our study did reveal that maternal smoke exposure was associated with lower infant 25(OH)D levels. These results support another study, which reported smoke exposure was also associated with lower vitamin D levels (Brot et al., 1999). Because of this strong association, minimizing maternal exposure to smoke may increase vitamin D levels. Since three-quarters of our mothers reported secondary smoke exposure and 7.6% of mothers reported a positive smoking history, supporting and enforcing public health interventions for smoking cessation would positive health benefits for both mothers and infants.

Limitations to our study exist, with the most significant limitation being that almost all of the infants in the study were vitamin D deficient, thus affecting our ability to make comparisons between those infants who are vitamin D deficient and those that are not deficient. Another limitation is that mothers were not monitored throughout their pregnancies. Additional information regarding vitamin D levels earlier in pregnancy would have provided insight into the specific groups most at risk for vitamin D deficiency and what factors contribute most to this risk. We also acknowledge that a universally accepted definition for vitamin D deficiency is controversial. A consensus has not yet been reached among experts in nutrition as to whether true vitamin D deficiency is less than 20 ng/mL or less than 11 ng/mL (Saintonge et al., 2009). Though some advocate for a threshold of less than 11 ng/mL, increasing that threshold to 20 ng/mL would result in substantially more individuals with vitamin D deficiency. Regardless, our population is considered deficient by both definitions. Additionally, cotinine levels were not measured to assess smoke exposure (Badran et al., 2009); however, the percent of smoke exposure is similar to other reports in this region (Miller et al., 2009; Ali et al., 2010; Khuri-Bulos et al., 2010). Another limitation is that the information gathered in the surveys is self-reported and supplementation intake was not verified with maternal medical records. We used liquid chromatography-tandem mass spectrometry to measure our vitamin D levels and reports of C-3 epimers of 25OHD(2) or 25OHD(3) have found in infants which can lead to overestimation of 25-OHD levels; however, our vitamin D levels are very low, so if anything, the vitamin D levels in our newborns could be even lower (Kamao et al., 2004; Singh et al., 2006). Of note, during the study period, there was interruption of the neonatal services due to expansion of obstetric and newborn services starting July 2010, which explains the varying number of births over the study period.

The high prevalence of extreme vitamin D deficiency in our large study population is troublesome and should alert clinicians to the necessity of proper nutritional supplementation, particularly in pregnant women. The Institute of Medicine of the National Academies (United States) recommends giving pregnant or lactating women 600 international units (IU) of vitamin D per day (up to 4,000 IU/day) to maintain an adequate vitamin D level. In addition, the implementation of vitamin D supplementation to the infant (400 IU/day) has the potential to significantly improve their vitamin D levels (Salle et al., 2001). Infants born during winter months are at particularly increased risk for developing hypovitaminosis D; however, mothers with estimated delivery dates during any period of the year should be targeted for vitamin D supplementation to ensure adequate vitamin D levels. The Jordanian government currently recommends adding vitamin D supplementation to wheat flour vitamin and mineral premix to help prevent vitamin D deficiency among the adult population in Jordan, and this may help to prevent vitamin D deficiency among pregnant Jordanian women and their infants. The prevention of vitamin D deficiency may play a major role in improving morbidity and mortality from pediatric illness such respiratory illnesses and other diseases. Moreover, ongoing studies of vitamin D levels surveillance, especially in women with childbearing potential and infants, and potential outcomes of vitamin D deficiency in this population are warranted.
